# Charged pyridinium oximes with thiocarboxamide moiety are equally or less effective reactivators of organophosphate-inhibited cholinesterases compared to analogous carboxamides

**DOI:** 10.1080/14756366.2022.2041628

**Published:** 2022-02-23

**Authors:** Zuzana Kohoutova, David Malinak, Rudolf Andrys, Jana Svobodova, Miroslav Psotka, Monika Schmidt, Lukas Prchal, Kamil Musilek

**Affiliations:** a Faculty of Science, Department of Chemistry, University of Hradec Kralove, Hradec Kralove, Czech Republic; b Biomedical Research Centre, University Hospital in Hradec Kralove, Hradec Kralove, Czech Republic

**Keywords:** Cholinesterase, organophosphate, oxime, inhibition, reactivation

## Abstract

The organophosphorus antidotes, so-called oximes, are able to restore the enzymatic function of acetylcholinesterase (AChE) or butyrylcholinesterase (BChE) via cleavage of organophosphate from the active site of the phosphylated enzyme. In this work, the charged pyridinium oximes containing thiocarboxamide moiety were designed, prepared and tested. Their stability and p*K_a_* properties were found to be analogous to parent carboxamides (K027, K048 and K203). The inhibitory ability of thiocarboxamides was found in low µM levels for AChE and high µM levels for BChE. Their reactivation properties were screened on human recombinant AChE and BChE inhibited by nerve agent surrogates and paraoxon. One thiocarboxamide was able to effectively restore function of NEMP- and NEDPA-AChE, whereas two thiocarboxamides were able to reactivate BChE inhibited by all tested organophosphates. These results were confirmed by reactivation kinetics, where thiocarboxamides were proved to be effective, but less potent reactivators if compared to carboxamides.

## Introduction

Organophosphates (OP) widely used as pesticides (e.g. chlorpyriphos, parathion) are synthetically prepared compounds that can also be misused as chemical weapons (e.g. nerve agents sarin, soman, tabun, VX)^1^. They are stable, and have a rapid effect, primary in the nervous system, after being absorbed by the skin and respiratory system^2^^,^^3^.

OP are irreversible inhibitors of ChEs, acetylcholinesterase (AChE, EC 3.1.1.7) and butyrylcholinesterase (BChE, EC 3.1.1.8). While inhibition of AChE can lead to life threatening intoxication due to cholinergic overstimulation and crisis, inhibition of BChE has no direct adverse effects. For this reason, BChE can be used as the OP bioscavenger and it is considered as a pseudo-catalytic bioscavenger^4^. The mechanism of AChE inhibition implies rapid phosphylation of the Ser203 hydroxyl group in AChE. After that AChE is no longer able to hydrolyse acetylcholine (ACh) which leads to its accumulation in the postsynaptic cleft followed by the overstimulation of cholinergic receptors^5^^,^^6^. Symptoms of poisoning are e.g. sweating, diarrhoea, tremors and muscle spasms. Death may be caused by respiratory system failure^6^.

**Figure 1.**
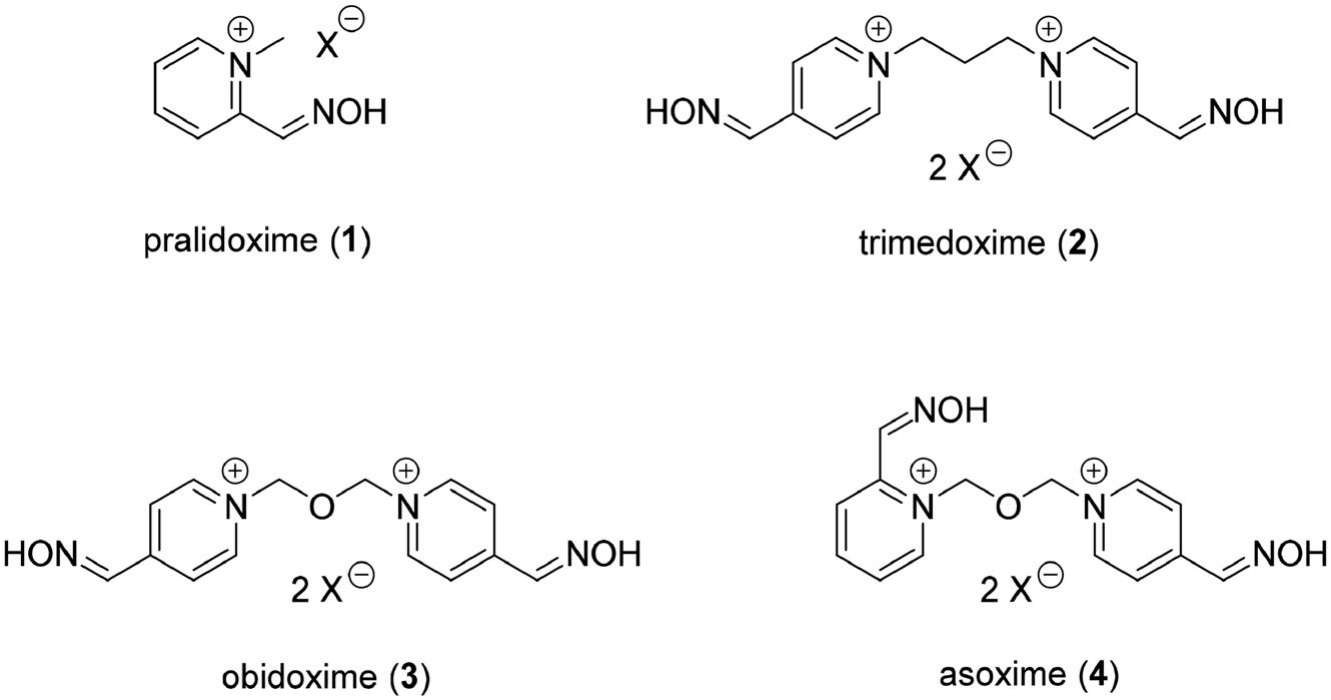
Structure of commercially available oxime reactivators.

Current medical countermeasures for therapy of intoxications caused by OPNAs consist of administration of atropine, oxime reactivator and anticonvulsant. While atropine and anticonvulsants are used for symptomatic therapy, oxime reactivators cleave the OP moiety from the active site of inhibited AChE and regenerates its function, therefore they serve as the causal antidotes^7^. Several oximes are already approved as antidotes against OPNA intoxication, i.e. pralidoxime (2-PAM) (**1**), trimedoxime (TMB-4) (**2**), obidoxime (**3**) and asoxime (HI-6) (**4**) (Figure 1)^8^^,^^9^.

**Figure 2.**
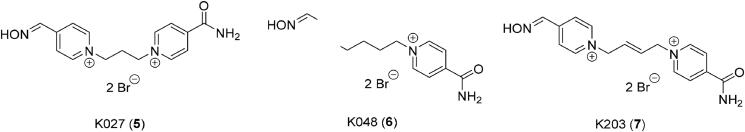
Structure of K027 (**5**), K048 (**6**) and K203 (**7**).

The major drawback of these reactivators is their limited broad-spectrum efficiency against various OPs and their low ability to pass the blood-brain barrier (BBB). That is why the current research is focussing on synthetising derivatives with modified properties, i.e. uncharged reactivators or modified nucleophiles^10–13^. Based on the structure of the standard monoquaternary and bisquaternary reactivators, novel oxime were prepared, e.g. derivatives of 2-PAM or group of reactivators called „K-oximes“^14–17^. Some of the K-oximes showed promising results in restoring activity of AChE inhibited by several OPs. I.e. K027 (**5**), K048 (**6**) and K203 (**7**) (Figure 2) have been presented to be potent reactivators of OP-inhibited AChE *in vitro* or *in vivo*^18–21^.

For this reason, the molecular design of charged pyridinium oximes with thiocarboxamide moiety was proposed from K027 (**5**), K048 (**6**) and K203 (**7**). The pyridinium scaffold with oxime moiety was retained as the α-nucleophile that is able to attack OP-inhibited cholinesterase. Similarly, the second aromatic moiety and the connecting linker remained to be same as in the parent molecules. Differently, the carboxamide moiety was replaced by thiocarboxamide to investigate the sulphur involvement in the binding of reactivator’s molecule within the active site of AChE or BChE. The carboxamide fragment was formerly found to play an important role in hydrogen bonding of charged oximes in the peripheral site of AChE^22^. For this reason, the use of spatially bulkier thiocarboxamide moiety could change such hydrogen bonding and overall affinity towards particular cholinesterase or it could increase the affinity to OP-enzyme complex for better reactivation.

In this work, the novel bis-pyridinium oximes were designed, synthetised and evaluated. The stability, oximate formation properties, inhibition of human ChEs and reactivation of human OP-inhibited ChEs was studied.

## Experimental

### Chemistry

All chemicals used for the synthesis were purchased from Sigma-Aldrich (Prague, Czech Republic) in the highest available purity. All used solvents were supplied by Penta Chemicals Unlimited (Prague, Czech Republic). Organophosphorus compounds 4-nitrophenyl isopropyl methylphosphonate (NIMP, sarin surrogate), 4-nitrophenyl ethyl methylphosphonate (NEMP, VX surrogate), 4-nitrophenyl ethyl dimethylphosphoramidate (NEDPA, tabun surrogate) and paraoxon (POX) were purchased from Chemforase (Mont-Saint-Aignan, France). Thin layer chromatography was performed on Merck silica gel 60 F_254_ and Merck cellulose F analytical plates. Detection was carried out with ultraviolet light (254 nm). Melting points were recorded on a Melting Point Apparatus - Büchi B-545 (Donau Lab, Czech Republic) without correction. The ^1^H and ^13^C NMR spectra were measured in DMSO-*d_6_* solution at room temperature on FT NMR spectrometer Avance NEO 500 MHz (499.87 MHz for ^1^H and 125.71 MHz for ^13^C) (Bruker, Germany). Chemical shifts, *δ*, are given in parts per million (ppm) and spin multiplicities are given as br s (broad singlet), s (singlet), d (doublet) or m (multiplet). Coupling constants, *J*, are expressed in hertz (Hz). For ^1^H δ is relative to DMSO-*d_6_* (*δ* = 2.50) and for ^13^C is relative to DMSO-*d_6_* (*δ* = 39.43). High Resolution Mass Spectrometry (HRMS) was determined by Q-Exactive Plus hybrid quadrupole-orbitrap spectrometer.

#### General procedure for synthesis of monoquaternary salts 8–10

The monoquaternary salts **8**–**9** were prepared by reaction of 4-hydroxymethylpyridine (32.8 mM) and dibromoalkane (163.8 mM) in acetone (30 ml). The mixture was stirred at reflux for 8 h, then cooled to room temperature and filtered and washed with acetone (3 × 20 ml). The crude product was crystalised from acetonitrile (100 ml per 1 g of product) at reflux and filtered under reduced pressure. The filtrate was evaporated under the reduced pressure to produce monoquaternary salts **8**–**9**^23^^,^^24^.

The monoquaternary salt **10** was prepared by reaction of 4-hydroxymethylpyridine (8.20 mM) and (*E*)-1,4-dibrombut-2-ene (40.90 mM) in acetone (30 ml). The mixture was stirred at reflux for 1.5 h, then cooled to room temperature and crystalline crude product was collected by filtration, washed with acetone (2 × 30 ml). The product was recrystalised from acetonitrile^25^.

#### General procedure for synthesis of bisquaternary salts 11–13

Secondly, the synthesis of the bisquaternary salt was completed. To a solution of monoquaternary salt **8**–**10** (1.48 mM) in dimethylformamide (DMF, 1.40 ml) was added 4-pyridinethioamide (2.22 mM). The resulting mixture was stirred at 60 °C for 48 h. The solvent was concentrated under the reduced pressure and the crude product was purified by crystallisation from acetonitrile at reflux. Then the solid was filtered, washed with acetonitrile and dried under the vacuum.

### 4-Carbamothioyl-1–(3-(4-((hydroxyimino)methyl)pyridinium-1-yl)propyl)pyridinium dibromide (11) K487

Compound **11** was isolated as orange solid, yield 245 mg (36%), m.p. 127.1–129.1 °C. ^1^H NMR (500 MHz, DMSO-*d_6_*): *δ* 2.65–2.71 (m, 2H, CH_2_), 4.75–4.80 (m, 4H, 2 × CH_2_), 8.28 (d, *J =* 6.1 Hz, 2H, 2 × ArH), 8.34 (d, *J =* 6.1 Hz, 2H, 2 × ArH), 8.47 (s, 1H, CH), 9.13 (d, *J =* 6.0 Hz, 2H, 2 × ArH), 9.24 (d, *J =* 6.1 Hz, 2H, 2 × ArH), 10.29 (br s, 1H, NH), 10.74 (br s, 1H, NH), 12.85 (s, 1H, OH). ^13^C NMR (126 MHz, DMSO-*d_6_*): *δ* 31.4, 56.9, 57.2, 124.1, 124.9, 145.0, 145.1 145.4, 148.5, 152.5, 194.1. HRMS (HESI^+^): [M + H]^2+^: calculated for C_15_H_18_N_4_OS^2+^ (m/2z): 151.0595; found: 151.0594.

### 4-Carbamothioyl-1–(4-(4-((hydroxyimino)methyl)pyridinium-1-yl)butyl)pyridinium dibromide (12) K488

Compound **12** was isolated as orange solid, yield 364 mg (52%), m.p. 180.3–182.3. ^1^H NMR (500 MHz, DMSO-*d_6_*): *δ* 1.96–2.02 (m, 4H, 2 × CH_2_), 4.67–4.76 (m, 4H, 2 × CH_2_), 8.25 (d, *J =* 6.3 Hz, 2H, 2 × ArH), 8.31 (d, *J =* 6.4 Hz, 2H, 2 × ArH), 8.45 (s, 1H, CH), 9.14 (d, *J =* 6.4 Hz, 2H, 2 × ArH), 9.25 (d, *J =* 6.4 Hz, 2H, 2 × ArH), 10.29 (br s, 1H, NH), 10.72 (br s, 1H, NH), 12.81 (s, 1H, OH). ^13^C NMR (126 MHz, DMSO-*d_6_*): *δ* 26.9, 27.0, 59.2, 59.5, 124.0, 124.9, 145.0, 145.2, 148.3, 152.4, 194.2. HRMS (HESI^+^): [M + H]^2+^: calculated for C_16_H_20_N_4_OS^2+^ (m/2z): 158.0673; found: 158.0673.

### 4-Carbamothioyl-1–(4-(4-((E)-(hydroxyimino)methyl)pyridinium-1-yl)but-2-en-1-yl)pyridinium dibromide (13) K489

Compound **13** was isolated as orange solid, yield 431 mg (61%), m.p. 199.1–201.1 °C. ^1^H NMR (500 MHz, DMSO-*d_6_*): *δ* 5.35–5.39 (m, 4H, 2 × CH_2_), 6.17–6.30 (m, 2H, 2 × CH), 8.27 (d, *J =* 6.7 Hz, 2H, 2 × ArH), 8.32 (d, *J =* 6.8 Hz, 2H, 2 × ArH), 8.46 (s, 1H, CH), 9.06 (d, *J =* 6.7 Hz, 2H, 2 × ArH), 9.17 (d, *J =* 6.8 Hz, 2H, 2 × ArH), 10.34 (br s, 1H, NH), 10.75 (br s, 1H, NH), 12.87 (s, 1H, OH). ^13^C NMR (126 MHz, DMSO-*d_6_*): *δ* 60.3, 60.6, 124.0, 124.9, 129.6, 130.6, 145.0, 145.1, 145.3, 148.7, 152.9, 194.2. HRMS (HESI^+^): [M + H]^2+^: calculated for C_16_H_18_N_4_OS^2+^ (m/2z): 157.0595; found: 157.0594.

### Stability determination

The stability of tested compounds was evaluated in two different media – demineralised water and phosphate buffered saline (PBS; Sigma-Aldrich P4417) as simulation of physiological environment. Compounds were dissolved in particular solvent (final concentration 1 mg/mL) and incubated at 37 °C. The amounts of analysed compounds were determined at time intervals of 0, 1, 2, 3, 4 and 5 h by UHPLC Infinity II 1290 system (Agilent Technologies, Santa Clara, USA) coupled with DAD detector. The stability was evaluated as the amount of remaining compound (%) in sample after specific time interval. GraphPad Prism version 8.2 (San Diego, USA) was used for the statistical data evaluation and visualisation.

### *pK*_a_ determination

The negative decimal logarithms of the dissociation constants (p*K_a_*) of prepared compounds were determined spectrophotometrically using buffers of given pH (range from 4.5 to 10.0 with 0.5 unit increment). Ten microliters of tested oxime (1 mg/mL) were dissolved in 490 µL of particular buffer and absorbance spectra of various dissociation states were scanned in range 200–400 nm using Carry-60 UV-VIS spectrophotometer (Agilent Technologies, Santa Clara, USA) at 20 °C. The p*K_a_* values were calculated from the sigmoidal dependence of the absorbance of the dissociated form of the substance on the pH value using GraphPad Prism 8.2 software (San Diego, USA).

### Inhibition assay

Recombinant forms of human acetylcholinesterase (*hr*AChE) and butyrylcholinesterase (*hr*BChE) were prepared at the Department of Chemistry, Faculty of Science, University of Hradec Kralove^26^. The inhibitory effect of tested oximes on *hr*AChE/*hr*BChE was determined by standard Ellman method adapted for 96-well plates^27^. The reaction mixture consists of *hr*AChE (final protein concentration 70 ng/mL) or *hr*BChE (220 ng/mL of protein), water solution of tested compound at appropriate concentration (the range of 1 µM to 500 µM for *hr*AChE and of 50 µM to 1500 µM for *hr*BChE) and solution of 5,5′-dithiobis-2-nitrobenzoic acid (DTNB, final concentration 500 µM, pH 7.4) in 20 mM Na-phosphate buffer. The mixture was pre-incubated for 15 min at 37 °C. Afterwards the substrate acetylthiocholine iodide (ATCI) or butyrylthiocholine iodide (BTCI) was added to the final concentration of 1000 µM. The total volume of reaction was 100 µL. Creation of the product 5-thio-2-nitrobenzoic acid (TNB), formed during the reaction was determined by observing its absorbance at specific wavelength 436 nm. The catalytic activity of enzyme was evaluated as amount of product (%) formed in the reaction after 10 min of incubation at 37 °C. IC_50_ values of compounds were calculated using non-linear regression by GraphPad Prism 8.2 (San Diego, USA). Data were calculated from three individual experiments which were made in triplicate.

### Reactivation screening

Recombinant enzymes (*hr*AChE/*hr*BChE) were inhibited by 25 µM POX, NEMP, NIMPor NEDPA for 30 min to obtain >99% inhibition. The excess of organophosphate was removed by dialysis against 25 mM Na-phosphate buffer (pH 7.4) for 16 h with three buffer exchanges. The tested oxime (10 µM or 100 µM) was incubated with inhibited enzyme for 15 or 30 min at 37 °C. The reaction mixture (100 µL) contained 10 µL of inhibited enzyme (1.95 ng of total protein), 20 µL of DTNB (2.5 mM), 10 µL of relevant oxime solution and 50 µL of Na-phosphate buffer (25 mM, pH 7.4). In parallel, reaction mixture containing no enzyme was followed as blank reaction to reflect oximolysis. The reaction was started by addition of 10 µL of substrate ATCI or BTCI (10 mM). The catalytic activity of enzyme reactivated by oxime was determined spectrophotometrically at specific wavelength 436 nm using Spark multimode microplate reader Tecan (Mannedorf, Switzerland). Data were calculated from three individual experiments which were made in triplicate.

### Reactivation kinetics

The selected compounds with promising reactivation ability were further tested to investigate reactivation kinetics parameters. Inhibited enzyme (*hr*AChE/*hr*BChE) was incubated for eight different times (0.5 to 15 min) with seven different concentrations of tested oxime (varying from 1 to 1200 µM) at 37 °C. As the blank the mixture containing no enzyme (to control oximolysis) was used. Acquired data were analysed by non-linear regression analysis according to Worek *et al*.^28^ using GraphPad Prism 8.2. Data were calculated from three individual experiments which were made in triplicate.

## Results and discussion

### Chemical synthesis

**Scheme 1.**
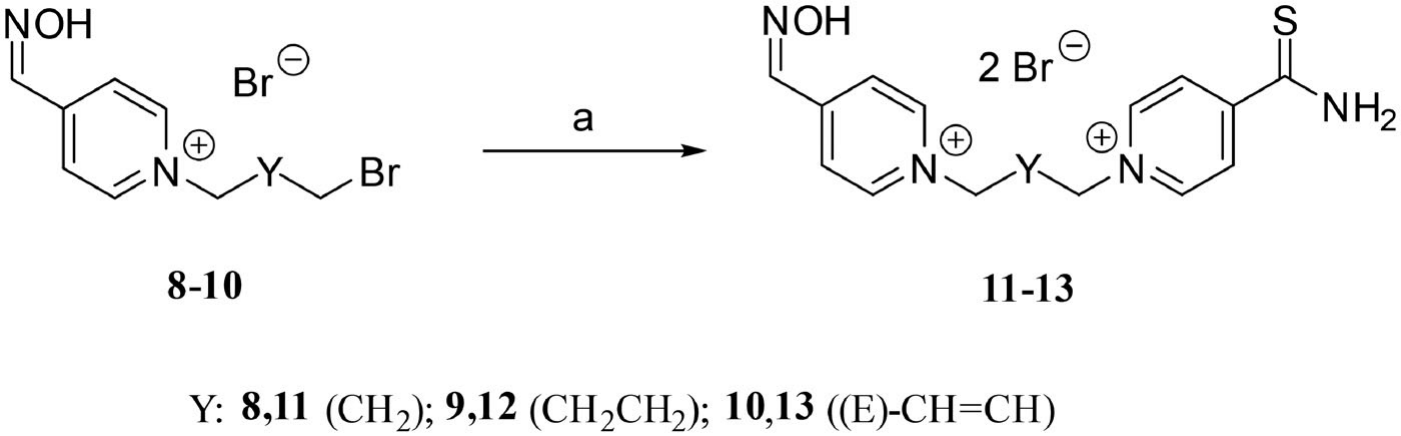
Synthesis of bisquaternary salts K487 (**11**), K488 (**12**) and K489 (**13**). Reagents and conditions: (a) 4-pyridinethioamide, DMF, 60 °C, 48 h, yields: 36% (for **11**), 52% (for **12**), 61% (for **13**).

The monoquaternary precursors **8**–**10** were formerly prepared by our research group^23–25^. Subsequent bimolecular nucleophilic substitution (S_N_2) of these monoquaternary salts with 4-pyridinethioamide in aprotic polar solvent DMF allowed creation of bisquaternary salts **11**–**13** (Scheme 1). The yields of these reactions ranged from 36% to 61%. The lowest yield was obtained in case of compound **11** (K487) which was probably caused by steric hindrance of two close pyridinium rings. The highest yield was obtained for compound **13** (K489), which contains but-2-en-1,4-diyl chain. In this case, the substrate **10** react rapidly by the S_N_2 mechanism because the π-system of the adjacent double bond can stabilise the transition state by conjugation. The final compounds were determined by NMR (Supplementary Figures 1–6) and HRMS analysis. Based on NMR and HRMS analysis, the non-calibrated purity of all products was ≥95%.

### Stability determination

**Figure 3.**
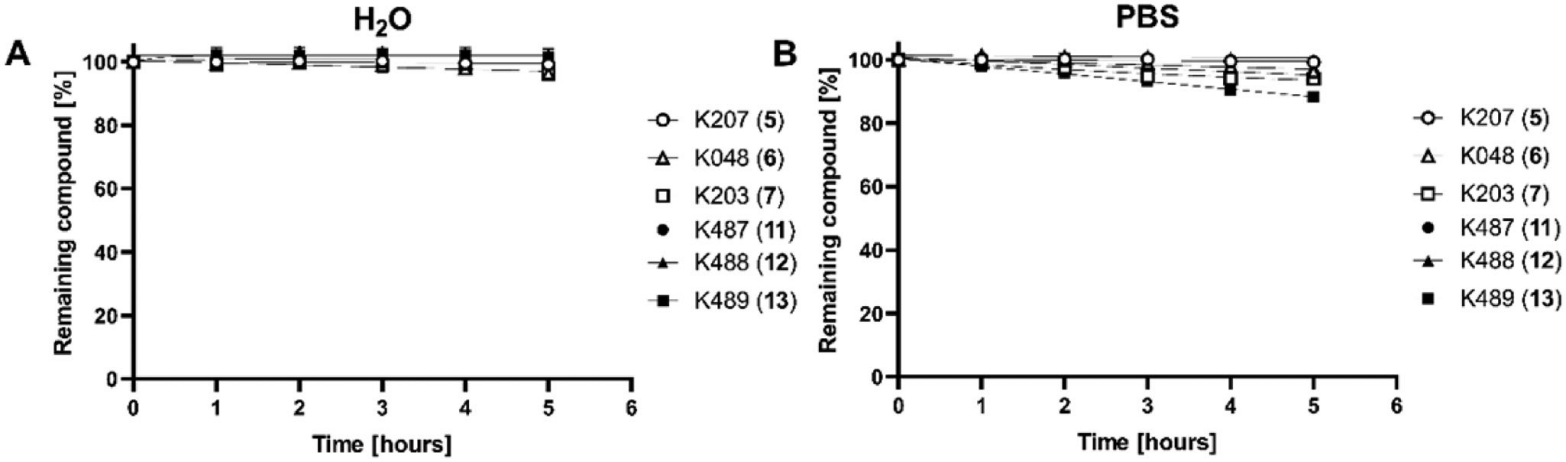
Stability of tested compounds in unionised water (**A**) and in PBS (**B**) at 37 °C.

Prepared compounds K487 (**11**), K488 (**12**) and K489 (**13**) and analogous oximes K027 (**5**), K048 (**6**) and K203 (**7**) were tested for stability in unionised water and PBS at 37 °C for 5 h, when the buffered environment and selected temperature are better mimicking human relevant conditions and the selected time is close to a full elimination of similar oximes from human organism. All compounds showed high stability in both media after 5 h (Figure 3). The degradation of tested oximes in pure water was found negligible. Some degradation of K489 (**13**) and K203 (**7**) was found in PBS buffer, but still with residual >90% oxime after 5 h. Stability is very important parameter for the compound use and also for maintaining the biological activity for the necessary time i.e. first 1–2 h after OP intoxication for charged oximes^29^.

### p*K_a_* determination

The p*K_a_* values were determined for compounds K487 (**11**), K488 (**12**), K489 (**13**), pralidoxime (**1**), asoxime (**4**) and for parent oximes (**5**–**7**). The p*K_a_* is an important parameter in pharmaceutical development to rationalise the physicochemical and biopharmaceutical properties of the drug molecule. It allows to detect protonated/deprotonated form of the compound under physiological conditions. In case of oximes deprotonated form so called oximate anion is important, because it is the active form of the oxime reactivator^30^. Change of amidic group into thioamidic group did not caused significant changes in the p*K_a_* values of oxime moiety (Table 1). This result was expected because sulphur does not differ much in electronegativity compared to oxygen. Moreover, thiocarboxamide moiety is far distanced to affect the electron density at the oxime pyridinium ring.

**Table 1.** The p*K_a_* values of tested compounds and parent oximes.

Compound	p*K_a_*
pralidoxime (**1**)	8.10 ± 0.01
asoxime (**4**)	7.27 ± 0.01
K027 (**5**)	8.18 ± 0.02
K048 (**6**)	8.18 ± 0.07
K203 (**7**)	8.12 ± 0.08
K487 (**11**)	8.14 ± 0.02
K488 (**12**)	8.22 ± 0.02
K489 (**13**)	8.13 ± 0.02

### In vitro enzyme inhibition

**Figure 4.**
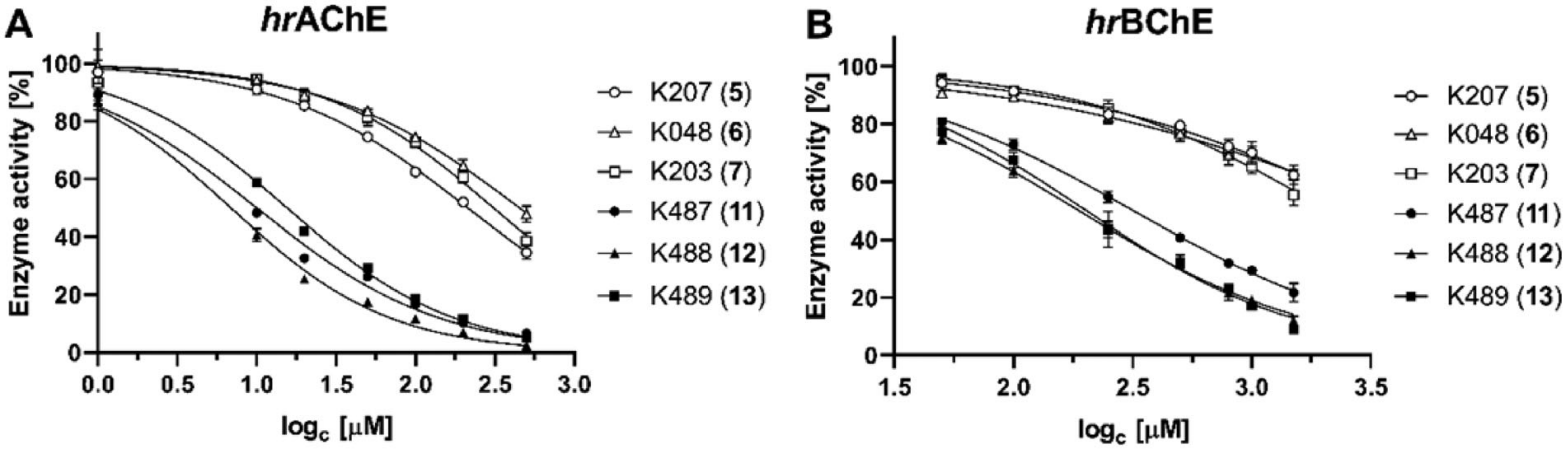
Inhibitory effect of tested compounds on *hr*AChE (**A**) and *hr*BChE (**B**).

All novel compounds **11**–**13** (K487-K489) and oximes K027 (**5**), K048 (**6**), K203 (**7**), pralidoxime (**1**) and asoxime (**4**) were tested for *in vitro* inhibition of *hr*AChE and *hr*BChE. While standard and parent oximes resulted as poor AChE and BChE inhibitors, compounds K487 (**11**), K488 (**12**) and K489 (**13**) resulted as relatively stronger inhibitors of both enzymes (Figure 4). The inhibition of *hr*AChE was found to be relatively higher in low µM scale (IC_50_^∼^7–16 µM), while inhibition of *hr*BChE resulted to be relatively lower in high µM scale (IC_50_^∼^189–320 µM). Oxime K488 (**12**) was found to be the most potent *hr*AChE (IC_50_^∼^7 µM) and *hr*BChE (IC_50_^∼^189 µM) inhibitor. This increase of inhibitory ability is most probably caused by sulphur atom. Although we did not study particular interaction with ChEs active sites, we suppose that bulkier sulphur atom has different binding and interactions at the active site gorge (e.g. hydrogen bonding properties) i.e. the sulphur atom is probably occupying more space within the peripheral site of the active site of AChE, which leads to stronger interactions with present aromatic amino acids and consequently increases the compound’s inhibition potency. The inhibition of BChE was less pronounced since BChE has less aromatic amino acids in its active site.

### In vitro reactivation screening

**Figure 5.**
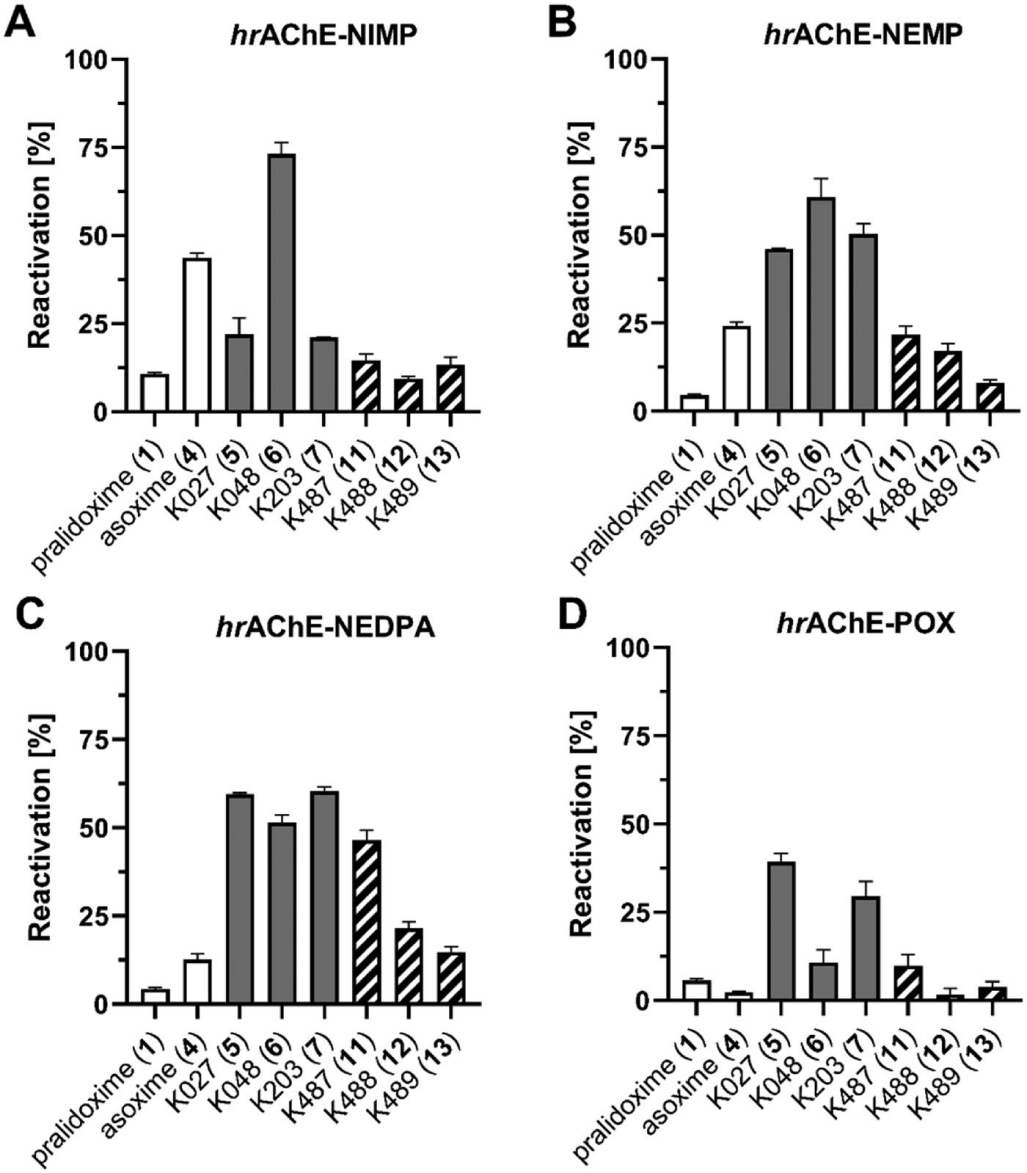
Reactivation of *hr*AChE inhibited by OP surrogates (%) with 10 µM oximes **11**–**13** after 15 min at 37 °C. Results were compared to pralidoxime (**1**), asoxime (**4**) and parent oximes (**5**–**7**).

All compounds were screened for reactivation ability on both ChEs inhibited by nerve agent surrogates NIMP, NEMP, NEDPA and POX^31^^,^^32^ as a representative of OP pesticides. The reactivation of OP-inhibited AChE was screened with 10 µM concentration of oxime after 15 min (Figure 5, Supplementary Table 2). For NIMP-AChE, the best reactivation was obtained for **6** (^∼^73%) followed by asoxime (**4**, ^∼^44%). Generally, the thiocarboxamides (**11–13**) were found as less potent reactivators when compared to carboxamide analogues (**5–7**). For NEMP-AChE, the best reactivation was observed with carboxamides (**5–7**, ^∼^46–61%) followed by thiocarboxamide K487 (**11**, ^∼^31%). The NEDPA-inhibited AChE was well reactivated by carboxamides (**5–7**, ^∼^51–60%) and thiocarboxamide K487 (**11**, ^∼^47%). The tested oximes were found to be poor reactivators of POX-inhibited AChE, and only K203 (**7**, ^∼^30%) was able to restore AChE activity to some extent.

Taken together, pralidoxime (**1**) and asoxime (**4**) were found to be generally less potent reactivators of NEMP-, NEDPA- and POX-inhibited AChE, when compared to carboxamides (**5–7**), while thiocarboxamides (**11–13**) resulted as weaker reactivators if compared to carboxamides. This result is most probably related to the stronger inhibition of AChE provided by thiocarboxamides which decreases overall activity of reactivated enzyme.

**Figure 6.**
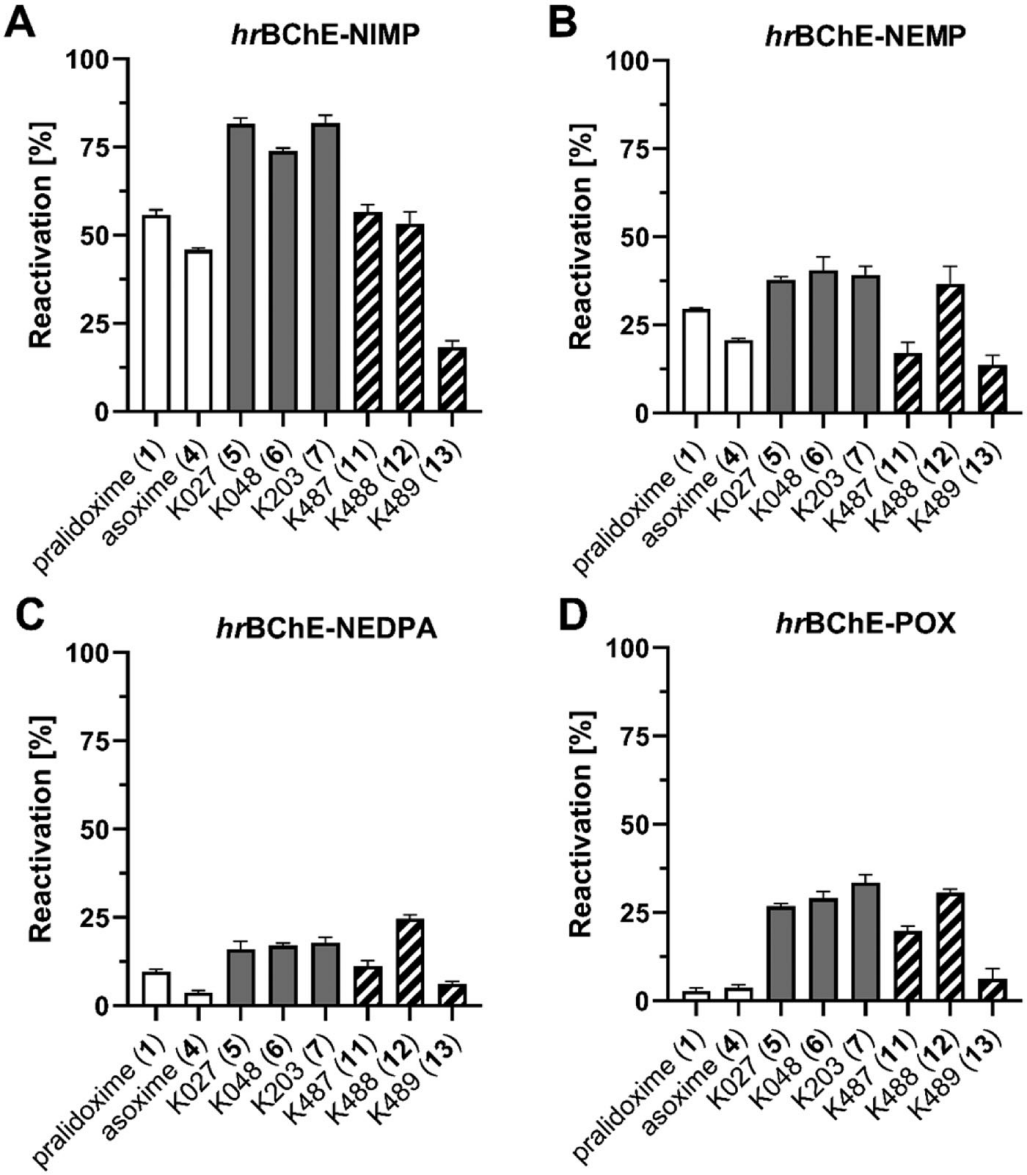
Reactivation of *hr*BChE inhibited by OP surrogates (%) with 100 µM oximes **11**–**13** after 15 min at 37 °C. Results were compared to pralidoxime (**1**), asoxime (**4**) and parent oximes (**5**–**7**).

The reactivation of OP-inhibited BChE was screened with 100 µM concentration of oxime after 15 min (Figure 6, Supplementary Table 3). In case of NIMP-BChE conjugate, carboxamides (**5–7**, ^∼^74–82%) were found as the best reactivators followed by thiocarboxamides (**11–12**, ^∼^53–57%) and pralidoxime (**1**, ^∼^56%). The most potent reactivators of NEMP-BChE conjugate were carboxamides (**5–7**, ^∼^38–41%)^33^ and thiocarboxamide **12** (^∼^37%) which was found to be the most potent reactivator of NEDPA-BChE as well. Surprisingly for NEDPA, thiocarboxamide **12** (^∼^25%) showed the best reactivation followed by carboxamides (**5–7**, ^∼^16–18%). The POX-BChE was reactivated almost equally by carboxamides (**5–7**, ^∼^27–34%) and thiocarboxamide **12** (^∼^31%). Taken together, pralidoxime (**1**) and asoxime (**4**) were found to be generally less potent reactivators of NIMP-, NEMP-, NEDPA- and POX-inhibited BChE, when compared to carboxamides (**5–7**) and equally or slightly less effective thiocarboxamide **12**. The better reactivation profile of thiocarboxamide **12** in case of BChE is related to the lower intrinsic inhibition of BChE and thus the overall higher activity of reactivated enzyme.

### Reactivation kinetics

For reactivation kinetics, only few thiocarboxamides with promising reactivation screening parameters were selected and their carboxamide analogues were chosen for comparison. Compound **11** was chosen for kinetic experiments in case of NEDPA-inhibited AChE, due to its highest reactivation ability in the screening and compounds **11–12** were selected in case of NIMP-inhibited BChE.

**Figure 7.**
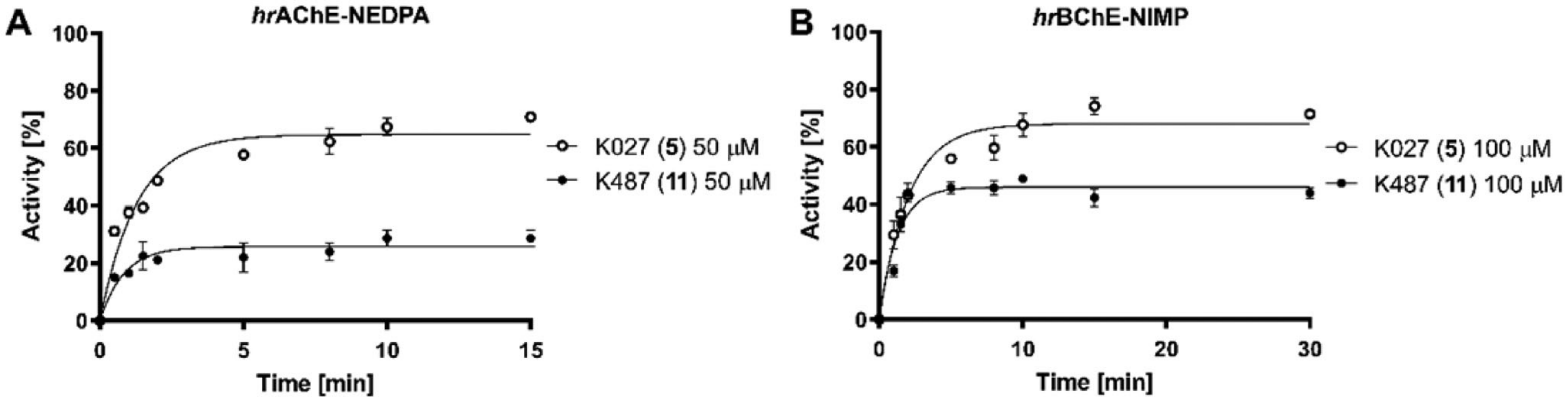
Reactivation kinetics of K027 (**5**) and K487 (**11**) of *hr*AChE-NEDPA (**A**) and *hr*BChE-NIMP (**B**).

The affinity of relevant oxime towards OP-inhibited recombinant enzyme (reflected by *K_D_*) and the ability to remove the OP residue from the active site of the enzyme (reflected by *k_r_*) were determined. Afterwards, the specific overall second-order reactivation rate constant (*k_r2_*) was calculated (*k_r2_*=*k_r_*/*K_D_*). The highest affinity for NEDPA-inhibited AChE, as well as the fastest dephosphorylation of catalytic serine (*k_r_*) was exhibited by oxime K027 (**5**), which therefore resulted with the highest *k_r2_* constant. Thiocarboxamide **11** was found to be a better reactivator of NEDPA-AChE than 2-PAM (**1**) and asoxime (**4**), but less effective than K027 (**5**) (Table 2). In addition, the reactivation kinetics of **5** and **11** towards NEDPA-AChE was directly compared by using 50 µM concentration of the reactivator (Figure 7). In this case, oxime **11** showed markedly lower reactivation which is caused by its stronger inhibition of AChE (IC_50_^∼^10 µM), which indicates more convenient use of carboxamide moiety instead of thiocarboxamide one when using the same (trimethylene) linker.

**Table 2.** Reactivation kinetic parameters of tested compounds.

Enzyme	OP	Oxime	Kinetic parameters		
			*K_D_* [µM]	*k_r_* [min^−1^]	*k_r2_* [mM^−1^min^−1^]
*hr*AChE	NEDPA	pralidoxime (**1**)^34^	51.89 ± 3.47	0.15 ± 0.07	2.9 ± 0.1
		asoxime (**4**)^34^	12.32 ± 1.18	0.55 ± 0.03	43.6 ± 1.9
		K027 (**5**)	7.70 ± 0.35	1.18 ± 0.07	155.8 ± 16.0
		K487 (**11**)	9.11 ± 0.68	1.01 ± 0.08	110.9 ± 17.1
*hr*BChE	NIMP	pralidoxime (**1**)^34^	415.60 ± 10.08	1.89 ± 0.03	4.6 ± 0.4
		asoxime (**4**)^34^	227.80 ± 5.71	1.05 ± 0.01	4.6 ± 0.1
		K027 (**5**)	37.80 ± 5.70	1.06 ± 0.02	28.0 ± 4.9
		K048 (**6**)	57.71 ± 3.51	0.67 ± 0.05	11.6 ± 1.6
		K487 (**11**)	32.81 ± 2.64	1.04 ± 0.03	31.7 ± 1.7
		K488 (**12**)	69.94 ± 6.40	0.55 ± 0.54	7.9 ± 1.6

NIMP-BChE conjugate showed the highest affinity for oxime K027 (**5**) and its analogue thiocarboxamide **11**. Furthermore, due to the high reactivation rate (*k_r_*) these oximes can be singled out as the best reactivators of NIMP-inhibited BChE with an overall reactivation rate of about 6-fold higher than 2-PAM and HI-6 (Table 2). The reactivation kinetics of **5** and **11** towards NIMP-BChE was also directly compared by using 100 µM concentration of the reactivator (Figure 7). Oxime **11** resulted as slightly weaker reactivator to its carboxamide analogue **5** which is again related to its slightly stronger inhibition of BChE.

The change of carboxamide into thiocarboxamide moiety doesn’t bring much benefit for reactivation of organophosphates and the presence of thiocarboxamide group causes unwanted higher inhibition of AChE/BChE and thus decreased reactivation.

## Conclusion

Three pyridinium mono-oximes containing thiocarboxamide group were designed, synthetised and tested. The novel compounds were evaluated for stability, and all compounds resulted as highly stable in both media (water and PBS). Further, p*K_a_* was determined and compared to parent oximes or standard compounds and no significant changes in the p*K_a_* values were found. The inhibitory effect of tested compounds on *hr*AChE and *hr*BChE was tested, and the determined IC_50_s were in the low µM range for *hr*AChE unlike for *hr*BChE. The novel compounds were evaluated for reactivation of *hr*AChE and *hr*BChE inhibited by NIMP (sarin-surrogate), NEMP (VX-surrogate), NEDPA (tabun-surrogate) and paraoxon. From reactivation screening, compound K487 (**11**) showed ability to reactivate AChE inhibited by NEMP and NEDPA. For BChE screening, K487 (**11**) and K488 (**12**) were found to have ability to reactivate all tested OPs. The reactivation kinetics was further determined for thiocarboxamides K487 (**11**) and K488 (**12**). Oxime **11** resulted as slightly weaker reactivator of NEDPA-AChE or NIMP-BChE if compared to its direct carboxamide analogue **5**, which is caused by its higher intrinsic inhibitory ability for both enzymes. Taken together, the tested thiocarboxamides resulted as equally or less effective charged oxime reactivators when compared to their carboxamide analogues. Therefore, the introduction of thiocarboxamide moiety doesn’t bring much benefit to the overall cholinesterase reactivation.

## Supplementary Material

Supplemental Material
