# Supplementary material for: Charged pyridinium oximes with thiocarboxamide moiety are equally or less effective reactivators of organophosphate-inhibited cholinesterases compared to analogous carboxamides
Source: J Enzyme Inhib Med Chem. 2022 Feb 23;37(1):760–7. doi: 10.1080/14756366.2022.2041628 (PMC8881075; doi:10.1080/14756366.2022.2041628)
Supplement: Supplemental Material [file IENZ_A_2041628_SM5166.pdf]

Supporting information

Charged pyridinium oximes with thiocarboxamide moiety are equally or less effective reactivators of organophosphate-inhibited cholinesterases compared to analogous carboxamides

Zuzana Kohoutova,[a] David Malinak,[a,b*] Rudolf Andrys,[a] Jana Svobodova,[a] Miroslav Psotka,[a,b] Monika Schmidt,[a,b] Lukas Prchal,[b] Kamil Musilek[a,b*]

[a] *University of Hradec Kralove, Faculty of Science, Department of Chemistry, Rokitanskeho 62, 500 03 Hradec Kralove, Czech Republic*

[b] *University Hospital in Hradec Kralove, Biomedical Research Centre, Sokolska 581, 500 05 Hradec Kralove, Czech Republic*

Content

1. **NMR spectra of final compouds 11-13** 2
2. ***In vitro* enzyme inhibition** 5
3. ***In vitro* reactivation screening** 5

1. NMR spectra of final compounds 11-13

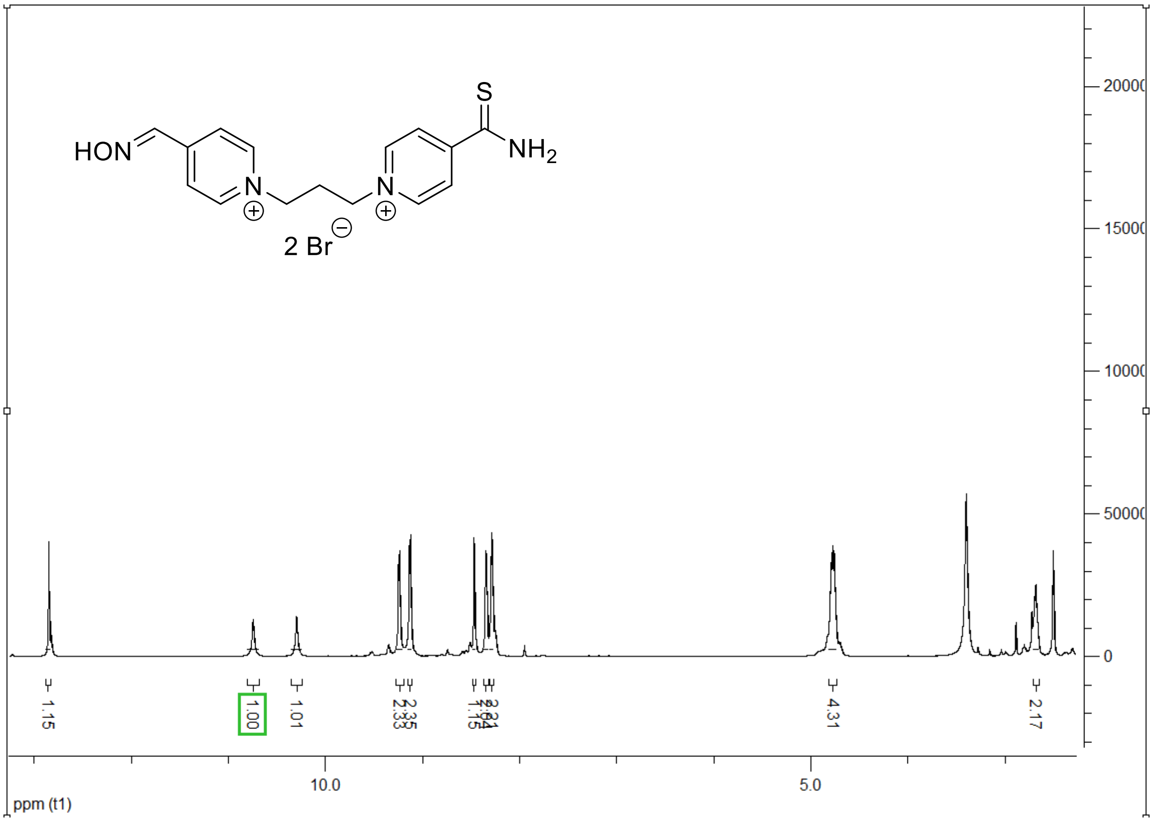

Figure S1. ^{1}H NMR spectrum of compound **11**.

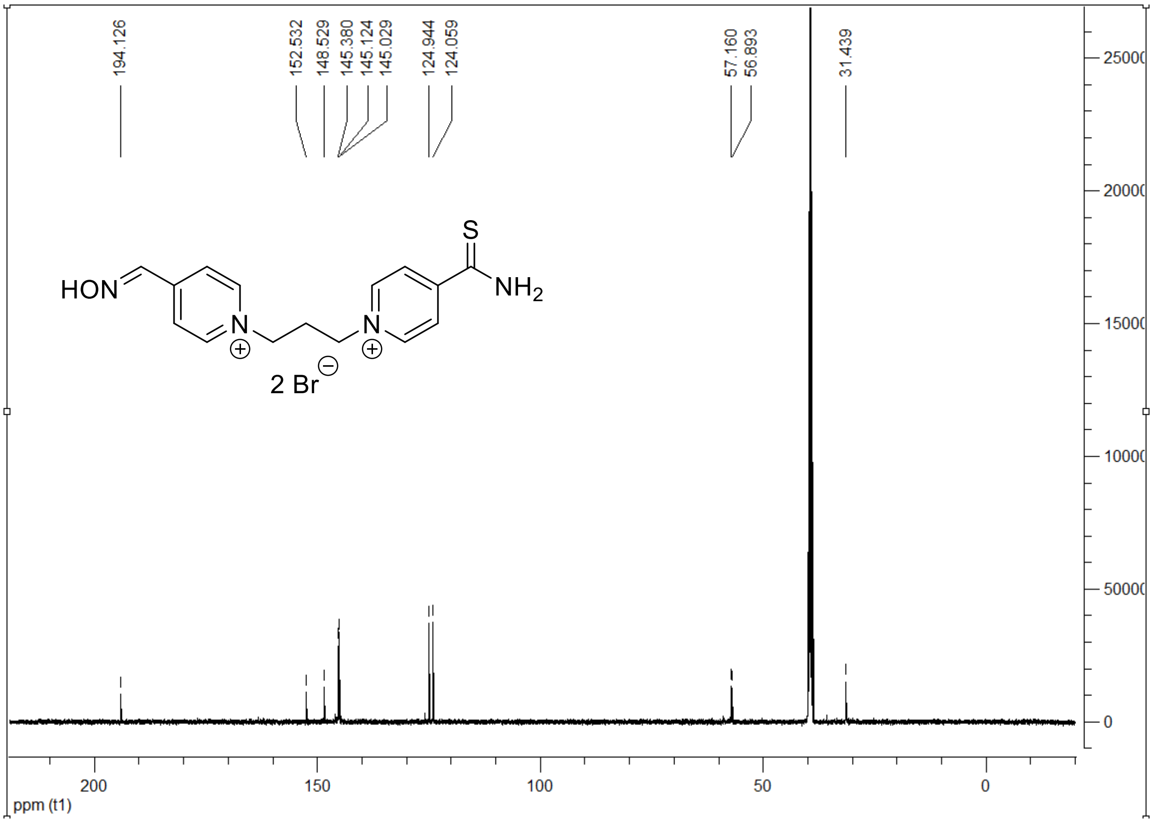

Figure S2. ^{13}C NMR spectrum of compound **11**.

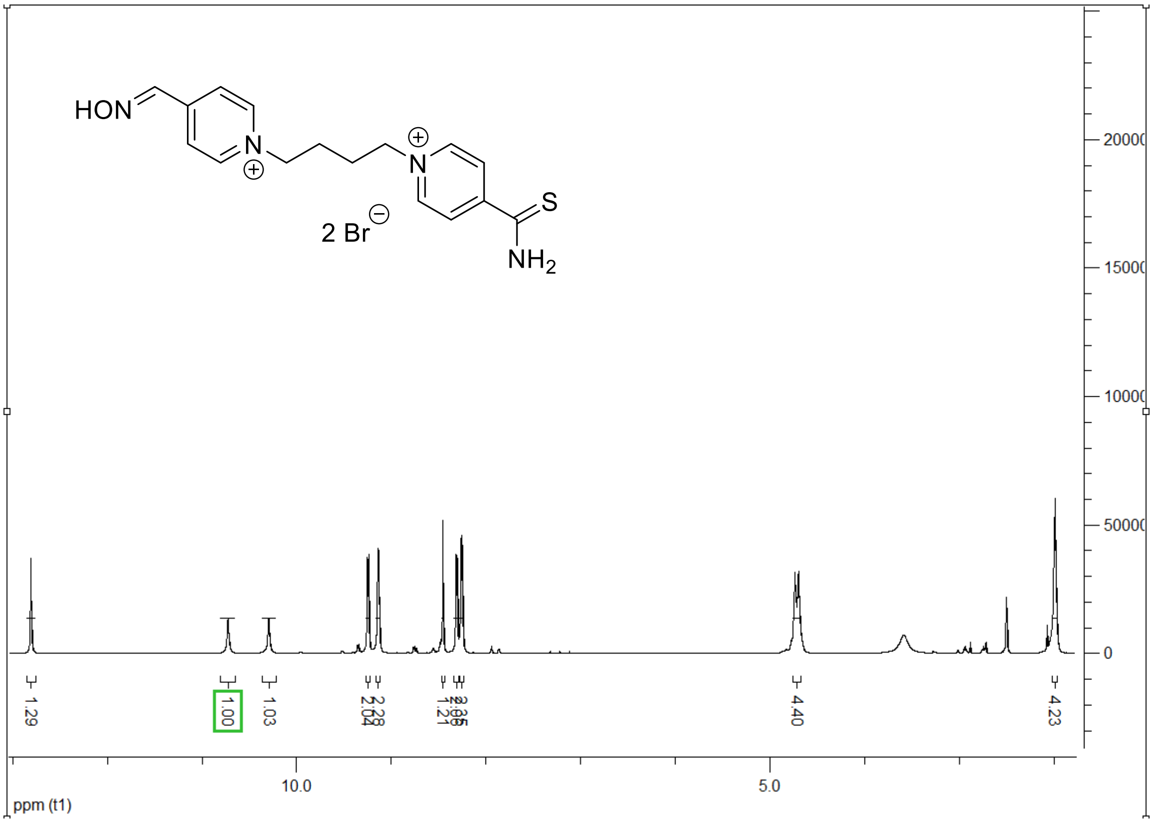

Figure S3. ^{1}H NMR spectrum of compound **12**.

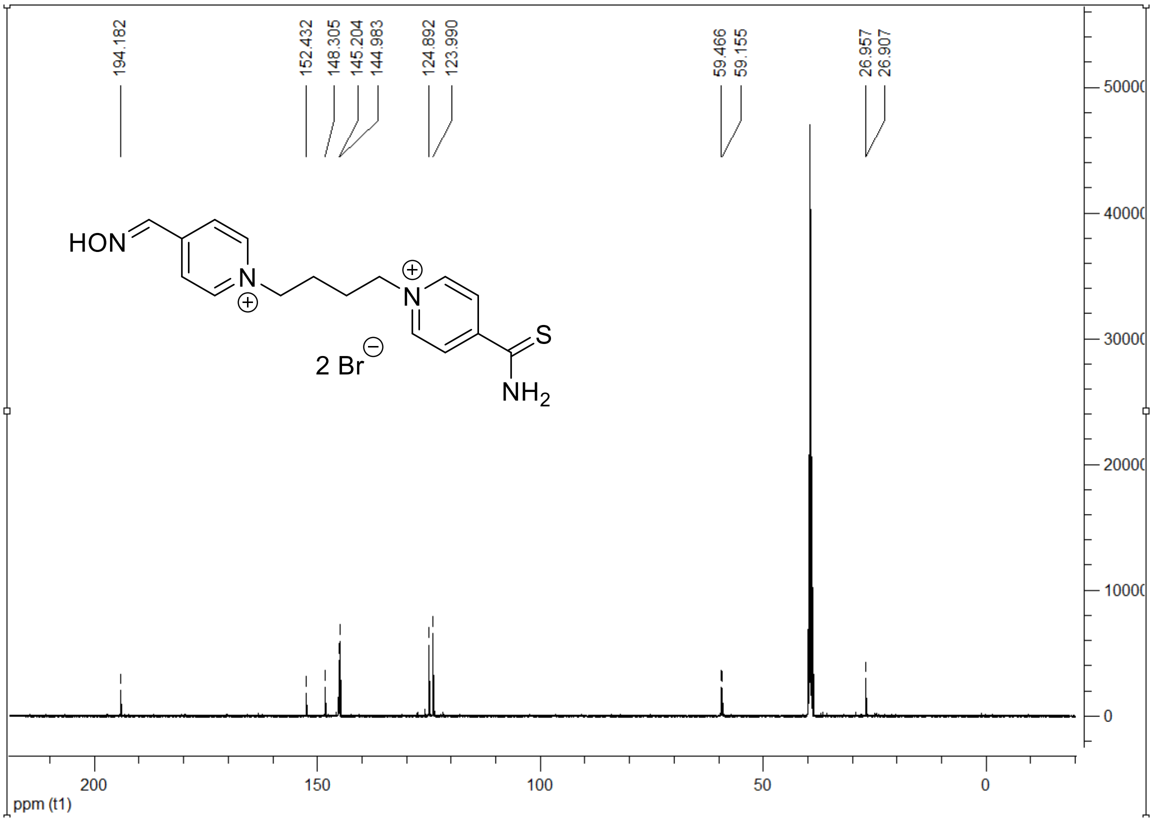

Figure S4. ^{13}C NMR spectrum of compound **12**.

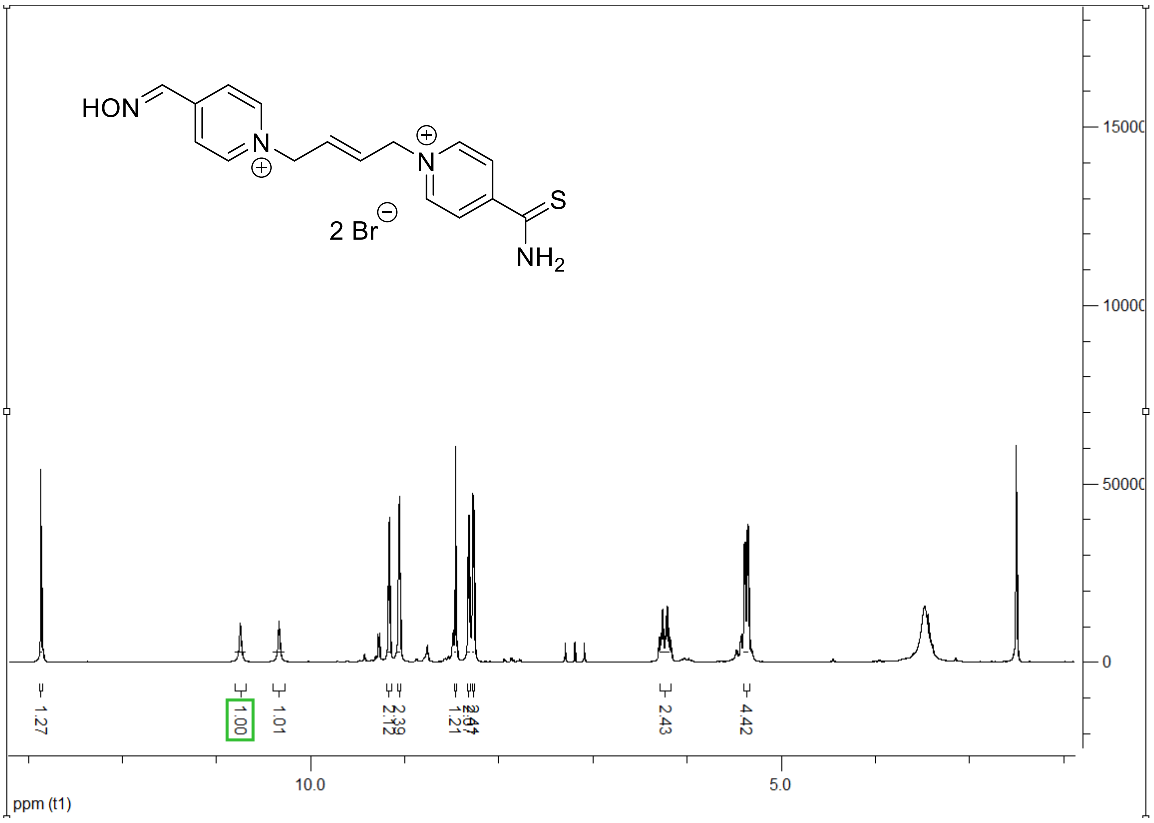

Figure S5. ^{1}H NMR spectrum of compound **13**.

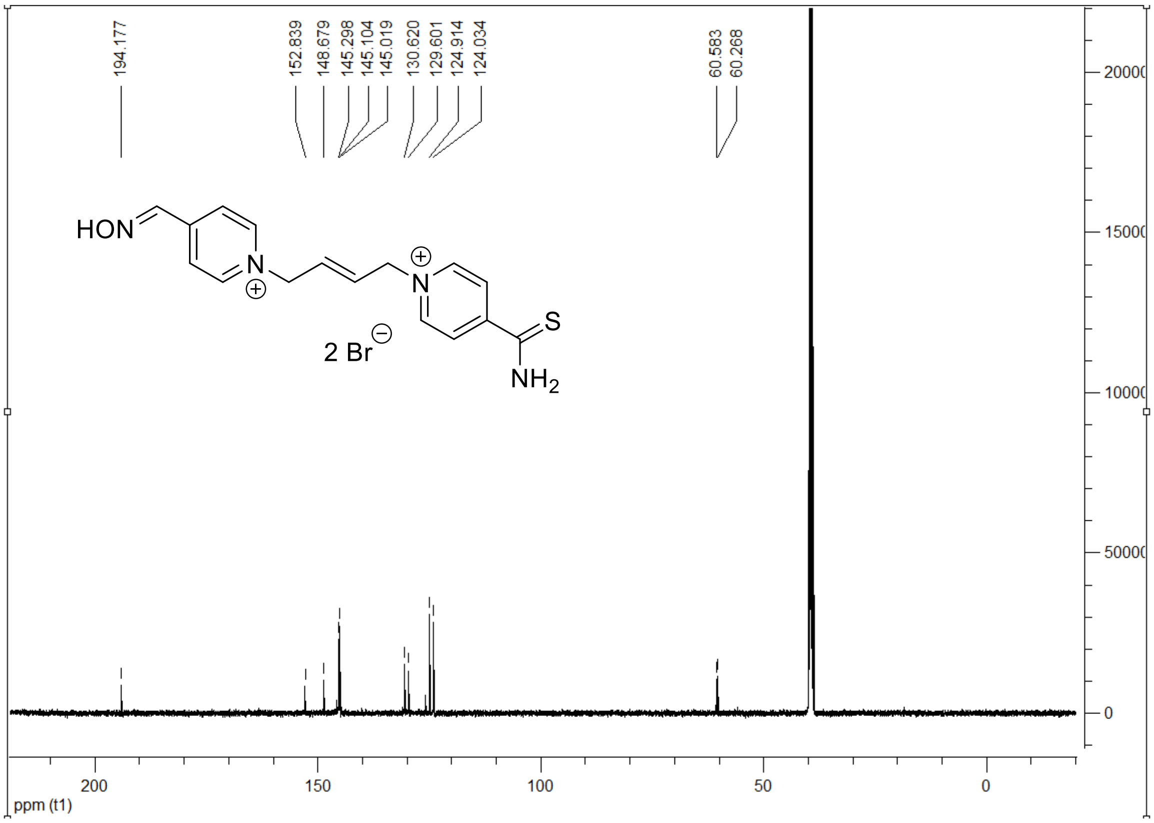

Figure S6. ^{13}C NMR spectrum of compound **13**.

2. *In vitro* enzyme inhibition

Table S1. Inhibition of cholinesterases by the tested compounds.

**Compound**	**IC_{50} *hr*AChE [μM]**	**IC_{50} *hr*BChE [μM]**
pralidoxime (**1**)	629.30 ± 25.67	> 3500
asoxime (**4**)	145.00 ± 7.08	1363 ± 48.17
K027 (**5**)	209.97 ± 4.84	> 1500
K048 (**6**)	476.53 ± 17.92	> 1500
K203 (**7**)	321.70 ± 13.73	> 1500
K487 (**11**)	9.99 ± 0.09	319.57 ± 21.02
K488 (**12**)	6.89 ± 0.68	188.97 ± 16.35
K489 (**13**)	15.51 ± 0.23	202.80 ± 23.10

3. *In vitro* reactivation screening

Table S2. Reactivation screening of standards and studied compounds on OP-inhibited *hr*AChE.

***hr*AChE**	**Reactivation [%]**			
	**NIMP**	**NEMP**	**NEDPA**	**POX**
pralidoxime (**1**)	10.7 ± 0.4	4.5 ± 0.4	4.3 ± 0.5	5.6 ± 0.5
asoxime (**5**)	43.7 ± 1.3	24.2 ± 1.2	12.6 ± 1.7	2.3 ± 0.3
K027 (**6**)	22.0 ± 4.6	46.1 ± 0.2	59.5 ± 0.4	39.3 ± 2.4
K048 (**7**)	73.1 ± 3.3	60.8 ± 5.2	51.5 ± 2.1	10.8 ± 3.6
K203 (**8**)	21.0 ± 0.1	50.3 ± 2.9	60.4 ± 1.2	29.6 ± 4.2
K487 (**12**)	14.6 ± 1.9	21.8 ± 2.4	46.5 ± 2.8	9.8 ± 3.2
K488 (**13**)	9.4 ± 0.7	17.1 ± 2.1	21.5 ± 1.8	1.6 ± 1.8
K489 (**14**)	13.3 ± 2.2	8.2 ± 0.7	14.8 ± 1.5	3.8 ± 1.6

Table S3. Reactivation screening of standards and studied compounds on OP-inhibited *hr*BChE.

***hr*BChE**	**Reactivation [%]**			
	**NIMP**	**NEMP**	**NEDPA**	**POX**
pralidoxime (**1**)	55.8 ± 1.4	29.5 ± 0.4	9.6 ± 0.7	2.9 ± 0.7
asoxime (**5**)	45.9 ± 0.5	20.7 ± 0.5	3.8 ± 0.5	3.8 ± 0.7
K027 (**6**)	81.5 ± 1.8	37.9 ± 0.7	16.1 ± 2.1	26.8 ± 0.7
K048 (**7**)	73.9 ± 0.8	40.5 ± 3.8	17.0 ± 0.7	29.0 ± 2.0
K203 (**8**)	81.8 ± 2.2	39.2 ± 2.5	17.9 ± 1.5	33.5 ± 2.3
K487 (**12**)	56.6 ± 2.1	17.0 ± 3.1	11.2 ± 1.7	19.8 ± 1.4
K488 (**13**)	53.3 ± 3.4	36.7 ± 4.9	24.7 ± 1.0	30.6 ± 1.0
K489 (**14**)	18.2 ± 1.9	13.7 ± 2.7	6.2 ± 0.6	6.3 ± 2.8
